# Assessment of Radiolabelled Derivatives of R954 for Detection of Bradykinin B1 Receptor in Cancer Cells: Studies on Glioblastoma Xenografts in Mice

**DOI:** 10.3390/ph17070902

**Published:** 2024-07-07

**Authors:** Miho Shukuri, Satoru Onoe, Tsubasa Karube, Risa Mokudai, Hayate Wakui, Haruka Asano, Shin Murai, Hiromichi Akizawa

**Affiliations:** Laboratory of Physical Chemistry, Showa Pharmaceutical University, Tokyo 194-8543, Japan

**Keywords:** bradykinin B1 receptor, R954, glioblastoma, nuclear imaging, molecular imaging

## Abstract

Bradykinin B1 receptor (B1R) has garnered attention as a cancer therapeutic and diagnostic target. Several reports on radiolabelled derivatives of B1R antagonists have shown favourable properties as imaging agents in cells highly expressing hB1R following transfection. In the present study, we assessed whether radiolabelled probes can detect B1R endogenously expressed in cancer cells. To this end, we evaluated ^111^In-labelled derivatives of a B1R antagonist ([^111^In]In-DOTA-Ahx-R954) using glioblastoma cell lines (U87MG and U251MG) with different B1R expression levels. Cellular uptake studies showed that the specific accumulation of [^111^In]In-DOTA-Ahx-R954 in U87MG was higher than that in U251MG, which correlated with B1R expression levels. Tissue distribution in U87MG-bearing mice revealed approximately 2-fold higher radioactivity in tumours than in the muscle in the contralateral leg. The specific accumulation of [^111^In]In-DOTA-Ahx-R954 in the tumour was demonstrated by the reduction in the tumour-to-plasma ratios in nonlabelled R954-treated mice. Moreover, ex vivo autoradiographic images revealed that the intratumoural distribution of [^111^In]In-DOTA-Ahx-R954 correlated with the localisation of B1R-expressing glioblastoma cells. In conclusion, we demonstrated that [^111^In]In-DOTA-Ahx-R954 radioactivity correlated with B1R expression in glioblastoma cells, indicating that radiolabelled derivatives of the B1R antagonist could serve as promising tools for elucidating the involvement of B1R in cancer.

## 1. Introduction

Kinins are a family of oligopeptides from the kallikrein–kinin system, generated in the inflammatory milieu of the tissue microenvironment, and are involved in numerous pathophysiological processes, including the regulation of blood pressure and inflammatory processes, pain sensation, and cell proliferation and migration [1,2]. Kinins signal through the activation of two G-protein-coupled receptors: inducible bradykinin receptor B1 (B1R) and constitutive receptor B2 (B2R). Activated kinin receptors in the cancer microenvironment are thought to be involved in tumour growth, angiogenesis, invasion, and cancer metastasis [3,4]. In particular, B1R is considered an important therapeutic target due to its inducible expression. However, it has been reported that the expression patterns and functions of B1R differ depending on the type of cancer. For instance, normal levels of B1R were observed in oesophageal carcinoma when compared with normal tissues, whereas increased levels of B1R characterise colorectal adenomas [5,6]. The function of B1R as a suppressor of cancer progression has been observed in melanoma cells [7], whereas its function as a modulator has been reported in colorectal, prostate, and breast cancers [8,9,10]. In studies of mice with intrastriatally implanted glioma cells, B1R selective antagonist-treated mice and B1R knockout mice showed a remarkable increase in tumour invasiveness, as indicated by tumour size or mitotic index [11]. However, combined treatment with B1R and B2R antagonists and B1R/B2R double knockout mice showed decreased tumour invasiveness, although pharmacological and genetic B2R blocking was not effective. Recently, stem cell-based therapies have gained popularity in the treatment of various diseases, including cancer. There are few reports on the role of B1R in the interactions between glioblastoma cells and mesenchymal stem cells, which are considered a promising source of stem cells for various therapies [12]. Thus, B1R in the cancer microenvironment represents an important therapeutic target, as it plays a role in regulating cancer progression. However, the efficacy of B1R regulation in cancer treatment remains unclear.

Imaging techniques capable of detecting B1R may be beneficial for investigating in vivo B1R changes in the cancer microenvironment. Nuclear medicine imaging techniques, such as positron emission tomography (PET) and single-photon emission computed tomography (SPECT), effectively allow for the noninvasive visualisation and measurement of physiological processes using radioactive molecular probes. Several reports have successfully developed PET probes that leverage B1R antagonist sequences [13,14,15,16,17,18]. [^68^Ga]Ga-DOTA-dPEG2-R954 (^68^Ga-HTK01083) and [^18^F]F-AmBF_3_-Mta-dPEG2-R954 (^18^F-HTK01146) are PET probes developed by Kuo et al. and are based on the properties of R954 (Ac-Orn-Arg-Oic-Pro-Gly-αMePhe-Ser-d-2-Nal-Ile), which is associated with favourable pharmacokinetics, stability, and safety profile [17]. PET studies with the probes conducted in mice bearing hB1R-expressing HEK293T tumours demonstrated high-contrast images of tumours, thus indicating that R954 is an effective lead sequence for the optimisation of B1R tracers for cancer imaging. Conversely, most of the previous probes targeting B1R have been evaluated using cells that highly expressed B1R following transfection. Aiming for clinical applications, it is important to clarify whether the probes can detect B1R expression levels comparable to those in patients. Thus, assessing this using cancer cell lines that endogenously express B1R could prove valuable.

To evaluate radiolabelled derivatives of B1R antagonists, we focused on the involvement of B1R in glioblastoma cells and considered using two typical glioblastoma cell lines (U87MG and U251MG), which are commonly used as experimental models of glioblastoma and have been reported to express B1R [19,20,21]. Glioblastoma is the most aggressive primary tumour of the central nervous system. It poses a significant challenge for effective therapy due to its high intra- and intertumoural heterogeneity, rapid invasion, and the presence of therapy-resistant subpopulations of glioblastoma stem cells and their inherent plasticity [22,23]. B1R promotes glioblastoma development by supporting the migration and invasion of glioblastoma cells [21]. Additionally, studies using co-culture models with bone marrow-derived mesenchymal stem cells showed an increase in B1R expression in U87MG cells, which was correlated with their enhanced invasiveness [24]. Thus, B1R as a potential regulator of glioblastoma has been demonstrated in some experiments, which regarded B1R as a potential biomarker for targeting in cancer therapy. Therefore, imaging techniques capable of detecting B1R are valuable for clinical research and basic glioblastoma research.

**Figure 1:**
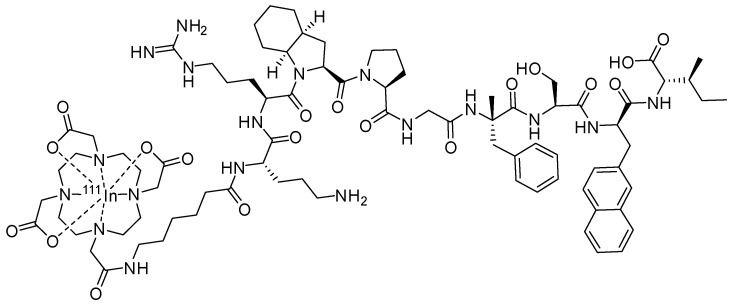
Chemical structure of [^111^In]In-DOTA-Ahx-R954.

In this study, we aimed to evaluate the ability of radiolabelled derivatives of a B1R antagonist to detect B1R in tumours derived from glioblastoma cell lines that endogenously express B1R. We synthesised ^111^In-labelled derivative of R954, in which the *N*-terminal acetyl group of R954 was replaced with aminohexanoic acid (Ahx) as a spacer, and 1,4,7,10-tetraazacyclododecane-1,4,7,10-tetraacetic acid (DOTA) was attached to the Ahx ([^111^In]In-DOTA-Ahx-R954) (Figure 1). We used [^111^In]In-DOTA-Ahx-R954 as an example of radiolabelled derivatives of the B1R antagonist based on previous studies that addressed the peptide antagonists of B1R, which are known to tolerate a certain level of *N*-terminal sequence modifications [25]. Furthermore, [^68^Ga]Ga-DOTA-dPEG2-R954, reported by Kuo et al., previously showed that R954 retains affinity for B1R after the introduction of DOTA to its *N*-terminus via a spacer [17]. To clarify the factors involved in the intratumoural distribution of [^111^In]In-DOTA-Ahx-R954, we conducted ex vivo autoradiography. We then compared the intratumoural distribution of blood flow as measured using [^14^C]iodoantipyrine, the expression pattern of B1R, and the presence of glioblastoma cells, as indicated by the glial fibrillary acidic protein (GFAP) marker expressed in astroglial tumours [26,27].

## 2. Results

### 2.1. In Vitro Accumulation of [^111^In]In-DOTA-Ahx-R954 in U87MG and U251MG Cells

**Figure 2:**
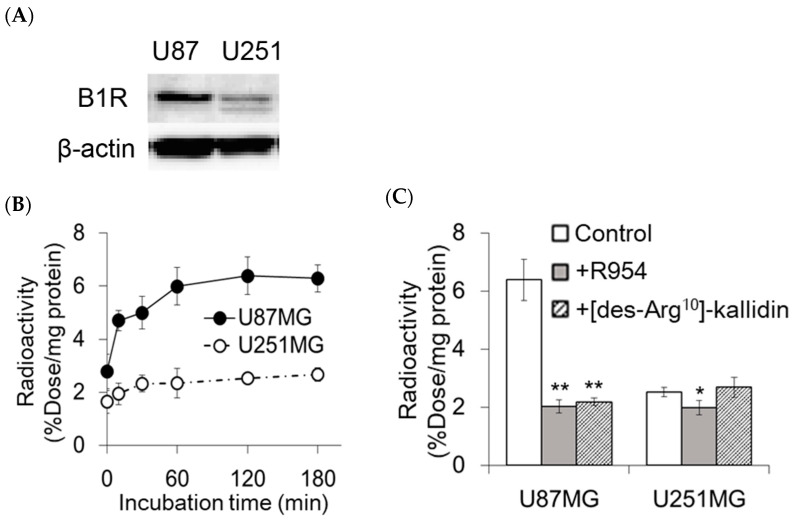
Specific accumulation of [^111^In]In-DOTA-Ahx-R954 in cultured U87MG and U251MG cells. (**A**) Immunoblotting of B1R; (**B**) Time-dependent changes in cell accumulated radioactivity following incubation with [^111^In]In-DOTA-Ahx-R954. Data are expressed as the mean ± SD (30 min in U87MG, *n* = 3, and in others, *n* = 4). *p* < 0.001 at all time points except 0 min, U87MG vs. U251MG; (**C**) Radioactivity of [^111^In]In-DOTA-Ahx-R954 in cells in the absence (control) or presence of 100 µM R954 (+R954) or [des-Arg^10^]-kallidin (+[des-Arg^10^]-kallidin). Data are expressed as mean ± SD (*n* = 4). * *p* < 0.01, ** *p* < 0.001, vs. control group (assay performed in the absence of antagonists and agonists).

**Figure 3:**
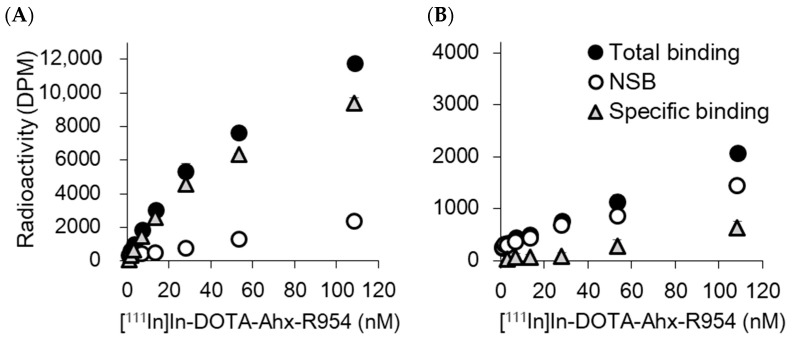
Binding of increasing concentrations of [^111^In]In-DOTA-Ahx-R954 to cultured U87MG and U251MG cells. Binding of [^111^In]In-DOTA-Ahx-R954 to U87MG (**A**) and U251MG (**B**) cells in the absence (total binding) or presence (nonspecific binding; NSB) of an excess (100 μM) of R954. Specific binding was obtained by subtracting NSB from total binding. Data are expressed as the mean ± SD (*n* = 4).

Immunoblotting revealed higher B1R expression levels in U87MG cells than in U251MG cells (Figure 2A and Figure S3). In U87MG cells, the accumulated radioactivity of [^111^In]In-DOTA-Ahx-R954 increased with an increase in the incubation time, reaching 6.29% dose/mg protein after 180 min of incubation (Figure 2B). U251MG cells showed no obvious time-dependent increase in accumulated radioactivity, and the accumulated level was significantly lower than that in U87MG cells at all time points. Nonlabelled R954 reduced the accumulated radioactivity in both cells. The decreased rates relative to the control group were 68.3% and 21.6% for the U87MG and U251MG cell lines, respectively (Figure 2C). In the blocking study with the B1R agonist [des-Arg^10^]-kallidin, a significant decrease In radioact”vity’was observed in U87MG cells but not in U251MG cells. Increasing concentrations of [^111^In]In-DOTA-Ahx-R954 were incubated with U87MG and U251MG cells in the presence or absence of nonlabelled R954 (Figure 3). In U87MG cells, the specific binding curve of [^111^In]In-DOTA-Ahx-R954 reached saturation, and Scatchard analysis demonstrated that [^111^In]In-DOTA-Ahx-R954 bound to a single class of sites with a K_d_ of 54.1 ± 18.9 nM and maximal binding (B_max_) of 0.95 ± 0.79 pmol/mg protein. Since the specific binding to U251MG cells was low and did not reach saturation over the range of concentrations examined, the K_d_ and B_max_ values for U251MG cells could not be determined.

### 2.2. Biodistribution of [^111^In]In-DOTA-Ahx-R954 in U87MG-Bearing Mice

First, we confirmed the plasma stability of [^111^In]In-DOTA-Ahx-R954 using C57BL/6JSlc male mice; 79.6 ± 3.1% of the radioactivity remained intact in the plasma 60 min after injection (Figure S4). Subsequently, in vivo radioactivity distributions in U87MG-bearing mice were assessed at several time points after injection (Table 1). At 1 h after injection, the accumulated radioactivity in the tumour engrafted in the right hind leg was 2.3-fold higher than that in the muscles of the nontreated left hind leg. Radioactivity levels in the plasma were higher than those in many tissues, including tumours, at 1 h after injection, and they gradually declined over time. At 4 h after injection, the decrease in radioactivity levels in the plasma (98% reduction compared to 1 h) resulted in decreased radioactivity levels in many tissues, including tumours (48% reduction compared to 1 h). Subsequently, the tumour-to-plasma ratios significantly increased at 4 h, and the highest ratio was observed at 24 h. Among the main organs, the kidneys exhibited the highest uptake, and the liver and lungs displayed relatively high radioactivity at 1 h after injection. Moreover, the liver, kidney, and large intestine showed increased radioactivity at 4 h, which was in contrast to the decreased radioactivity in most other organs at this time point. At 24 h, the radioactivity in the kidney remained high at 109.2% dose/g tissue.

To hamper the specific accumulation of [^111^In]In-DOTA-Ahx-R954 in vivo, an excess of nonlabelled R954 was administered to U87MG-bearing mice (Table 2). Simultaneous administration of nonlabelled R954 significantly decreased the accumulation of [^111^In]In-DOTA-Ahx-R954 in the kidneys at both 1 h and 4 h after injection (60% and 17% decrease, respectively). However, other tissues, including tumours, showed increased R954-induced radioactivity at 1 h after injection. The highest rate of increase was shown in plasma (374%), and the next highest rates were in the muscle (324%) and stomach (315%) tissues. These results suggest that R954 induced an increase in plasma concentrations of [^111^In]In-DOTA-Ahx-R954, resulting in increased radioactivity concentrations in all tissues except the kidneys. At 4 h after injection, although plasma concentrations in the R954 treatment group decreased compared to 1 h after administration, they remained higher than those in the control group. In most organs except the kidneys, the radioactivity levels of R954-treated mice were comparable to those of the control group. When comparing the plasma concentration ratios, we observed that the R954-treated group showed significant decreases in tumour-to-plasma ratios (30% and 69% reduction at 1 h and 4 h after injection, respectively), indicating the specific accumulation of [^111^In]In-DOTA-Ahx-R954 in tumours.

### 2.3. Ex Vivo Autoradiography of [^111^In]In-DOTA-Ahx-R954 and [^14^C]iodoantipyrine

**Figure 4:**
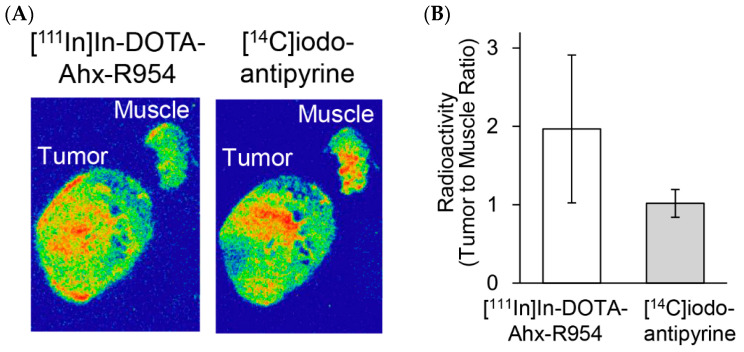
Intratumoural distribution of [^111^In]In-DOTA-Ahx-R954 and [^14^C]iodoantipyrine. (**A**) Representative ex vivo autoradiographs of [^111^In]In-DOTA-Ahx-R954 (60 min post-injection) and [^14^C]iodoantipyrine (1 min post-injection) obtained from the same slide; (**B**) Radioactivity data (tumour-to-muscle ratio) in the ex vivo autoradiographs are expressed as mean ± SD (in [^111^In]In-DOTA-Ahx-R954, *n* = 5, and in [^14^C]iodoantipyrine, *n* = 4).

A blocking study using R954 in mice revealed that the plasma concentration of [^111^In]In-DOTA-Ahx-R954 had a significant influence on tissue accumulation. To clarify the effect of blood flow on [^111^In]In-DOTA-Ahx-R954 accumulation in tumours, we compared the intratumoural distribution of [^111^In]In-DOTA-Ahx-R954 and [^14^C]iodoantipyrine, a blood flow tracer, visualised using ex vivo autoradiographic images obtained via the double tracer method. Representative ex vivo autoradiographs of [^111^In]In-DOTA-Ahx-R954 and [^14^C]iodoantipyrine on identical slides showed different accumulation distributions in the tumour and muscle tissues (Figure 4A). In addition, the [^14^C]iodoantipyrine data revealed that blood flow did not change between the tumour and muscle, indicating that the higher accumulation of [^111^In]In-DOTA-Ahx-R954 in tumours compared to muscles under normal conditions was not dependent on changes in blood flow (Figure 4B).

### 2.4. B1R Expression and Intratumoural Distribution of Glioblastoma Cells

**Figure 5:**
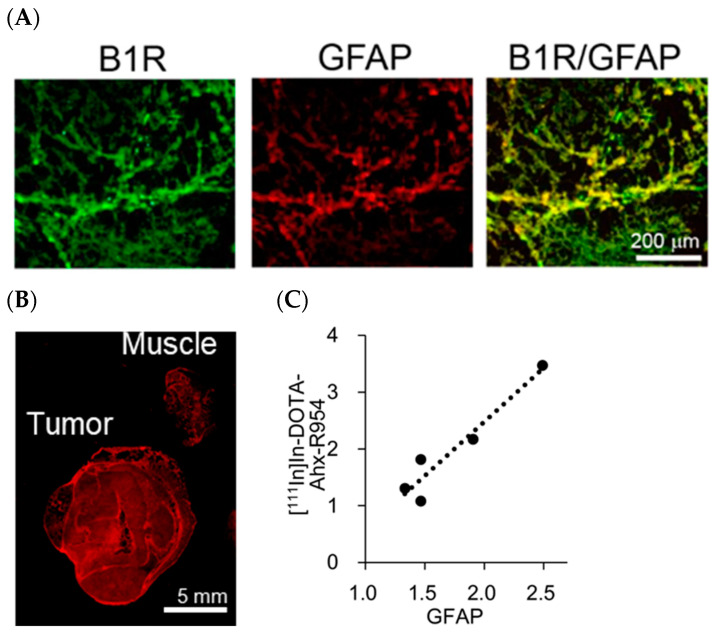
B1R expression and intratumoural distribution of GFAP-positive glioblastoma cells. (**A**) Representative photomicrographs of double immunofluorescent labelling for B1R (green) and GFAP (red) in U87MG tumour tissue; (**B**) Representative macro image of GFAP immunoreactivity detected using an Amersham™ Typhoon™ scanner; (**C**) Correlation between the radioactivity of [^111^In]In-DOTA-Ahx-R954 and the fluorescence intensity of GFAP. The analysis was evaluated using the value of the tumour-to-muscle ratio. The correlation coefficient was 0.96.

Figure 5A shows representative photomicrographs of the U87MG tumour tissue double immunofluorescence labelled for B1R and GFAP, where [^111^In]In-DOTA-Ahx-R954 showed high accumulation. In this field of view, B1R-positive cells colocalised with GFAP-positive glioblastoma cells. The macro images of GFAP immunoreactivity showed higher fluorescence intensity in the tumour than in the muscle (Figure 5B). Compared to the ex vivo autoradiography of [^111^In]In-DOTA-Ahx-R954 from the same object shown in Figure 4A, the intratumoural distribution of GFAP-positive cells was consistent with the [^111^In]In-DOTA-Ahx-R954 accumulated distribution. The tumour-to-muscle ratio of the analysed fluorescence intensity in the images of GFAP-positive cells correlated well with the [^111^In]In-DOTA-Ahx-R954 accumulation (Figure 5C).

## 3. Discussion

In the present study, we evaluated whether [^111^In]In-DOTA-Ahx-R954, a radiolabelled derivative of the B1R antagonist, could detect B1R endogenously expressed in glioblastoma cells. Cellular uptake experiments in two cell types with different B1R expression levels, U87MG and U251MG, suggest that the accumulation rate of [^111^In]In-DOTA-Ahx-R954 reflects the differences in B1R expression levels. U87MG cells showed a higher expression of B1R and an accumulation of [^111^In]In-DOTA-Ahx-R954 compared with U251MG cells. The blocking effects of the nonradiolabelled B1R antagonist R954 and agonist [des-Arg^10^]-kallidin on [^111^In]In-DOTA-Ahx-R954 accumulation were also higher in U87MG than in U251MG cells, indicating that the accumulation of [^111^In]In-DOTA-Ahx-R954 is specific to B1R in glioblastoma. Scatchard analysis from the saturation experiment revealed a single class of binding sites in U87MG cells with a K_d_ of 54.1 ± 18.9 nM and B_max_ of 0.95 ± 0.79 pmol/mg protein.

Based on the favourable results observed in vitro, we evaluated [^111^In]In-DOTA-Ahx-R954 under in vivo conditions using U87MG tumour-bearing mice. The engrafted tissue in U87MG tumours demonstrated a two-fold higher accumulation of [^111^In]In-DOTA-Ahx-R954 compared to the muscle in the non-treated leg. The tumour-to-plasma ratios were increased with decreasing plasma concentration in a time-dependent manner after injection of [^111^In]In-DOTA-Ahx-R954. The value observed at 4 h was comparable to the tumour-to-blood ratio of [^68^Ga]HTK01083 and [^18^F]HTK01146 at 1 h post injection [17]. However, in a blocking study conducted in mice, simultaneous injection of R954 increased the accumulation rate in most organs, including tumours, at 1 h after injection. This was mainly driven by an increased plasma concentration of [^111^In]In-DOTA-Ahx-R954 induced by R954. In a previous report on [^68^Ga]HTK01083, elevations in radioactivity were observed in organs other than the kidneys and tumours derived from B1R-expressing HEK293T cells established through transfection [17]. Based on the increased radioactivity in the blood, the authors suggested that the dosage of the administered blocking agent may have saturated the clearance pathways. In our study, conducted under the same administration conditions, R954 significantly decreased the accumulation rate in the kidneys, thus indicating that the renal route, as the main excretion route of [^111^In]In-DOTA-Ahx-R954, was saturated. Even after 4 h, most organs, except for the kidneys, in the R954 treatment group showed accumulation levels equivalent to those in the control group, suggesting that increased plasma concentration caused nonspecific accumulation. Thus, to correct for the effect of increased plasma concentration, we re-assessed the blocking effect of R954 using the ratio to plasma concentration. Consequently, the corrected accumulation rate in R954-treated mice decreased in most organs. Furthermore, our results indicate reductions in the tumour-to-plasma ratios in R954-treated mice (30% and 69% reduction at 1 h and 4 h after injection, respectively), emphasising the specific accumulation of [^111^In]In-DOTA-Ahx-R954 under in vivo conditions. In addition, the decrease in the corrected accumulation rate in organs, including the heart, lungs, liver, and kidneys, further supports the specificity of [^111^In]In-DOTA-Ahx-R954 against B1R. This is consistent with previous reports of B1R expression using quantitative real-time RT-PCR of B1R mRNA in mice [28,29].

Our blocking experiments using R954 in mice revealed that the plasma concentration of [^111^In]In-DOTA-Ahx-R954 had a significant influence on tissue accumulation. Pharmacokinetics in tumours have been reported to be influenced by tumour blood flow [30]. Therefore, to elucidate the effect of blood flow on [^111^In]In-DOTA-Ahx-R954 accumulation in tumours, we compared the intratumoural distribution of [^111^In]In-DOTA-Ahx-R954 relative to the blood flow, determined using the [^14^C]iodoantipyrine marker. Ex vivo autoradiographic images obtained using the double tracer method revealed that the intratumoural distribution of [^111^In]In-DOTA-Ahx-R954 was not dependent on the local blood flow in the tumour. In addition, no difference was found in blood flow between tumours and muscles, further indicating that the increase in tumour accumulation of [^111^In]In-DOTA-Ahx-R954 was not affected by angiogenesis-induced changes in blood flow. Immunohistochemical studies demonstrated the colocalisation of B1R with GFAP, a marker for glioblastoma, in the area where accumulated radioactivity was observed in the autoradiogram with [^111^In]In-DOTA-Ahx-R954. These results indicate that radiolabelled R954 can detect B1R endogenously expressed in the U87MG glioblastoma cell line, which is valuable for understanding the role of B1R in cancer.

Our study has several potential limitations. First, the binding affinity of [^111^In]In-DOTA-Ahx-R954 (K_d_ = 54.1 ± 18.9 nM for U87MG cells) was lower than that of previously reported radioligands for B1R, such as [^3^H](des-Arg^10^, Leu^9^)-kallidin (0.33 ± 0.07 nM and 1.9 ± 0.5 nM for lung fibroblast cell membrane and B1R+ HEK293T cell membrane, respectively) [13,31]. Although we could not evaluate the diagnostic utility of [^111^In]In-DOTA-Ahx-R954 by comparing it with previously reported probes for SPECT and PET using parameters obtained under the same experimental conditions, improving the affinity is important in the development of [^111^In]In-DOTA-Ahx-R954 as a diagnostic agent. Additionally, the poor blood–brain barrier penetration of [^111^In]In-DOTA-Ahx-R954 makes it difficult to use for detecting tumours present in the brain. To further develop SPECT probes targeting B1R, small molecule agonists and antagonists targeting B1R [18,32,33,34] may be useful as lead compounds for B1R imaging probes due to their improved blood–brain barrier penetrance. Second, the number of glioblastoma cell lines used (U87MG and U251MG) was limited. Although the two cell lines were used as a common model for glioblastoma, using more types of cells that have different B1R expression levels is necessary to clearly determine the characteristics of [^111^In]In-DOTA-Ahx-R954. Additionally, as bradykinin receptors in glioma cells have been reported to be positively correlated with the World Health Organization tumour grade [11,35], it is important to clarify the correlation between [^111^In]In-DOTA-Ahx-R954 accumulation and grade. Moreover, the utilisation of a radiolabelled B1R antagonist for studies using glioma stem cell models and coculture systems with mesenchymal stem cells is required for further advancement of research on stem cell-based therapies for glioblastoma.

In summary, the present study demonstrated that [^111^In]In-DOTA-Ahx-R954 could detect B1R in tumours derived from endogenous B1R-expressing U87MG glioblastoma cells. Nevertheless, the assessment of [^111^In]In-DOTA-Ahx-R954 as a SPECT probe should be approached with caution, considering its high accumulation in normal tissues, such as the kidneys, and the unsatisfactory tumour-to-background contrast. However, the finding that the accumulation of [^111^In]In-DOTA-Ahx-R954 is positively correlated with tumour B1R expression levels suggests potential clinical applications for radiolabelled derivatives of B1R antagonists, which have been actively studied as PET/SPECT probes. Moreover, these probes can be valuable for evaluating models with B1R expression patterns similar to those observed in patients. While our experiments were conducted using glioblastoma cells as an example, B1R-targeting probes have broad applications across various cancer types. The expression patterns and functions of B1R differ depending on the type of cancer [5,6,7,8,9,10]. Moreover, developing radiolabelled probes using a B1R antagonist holds potential in elucidating the function of B1R in cancers.

## 4. Materials and Methods

### 4.1. Synthesis

Fmoc-d-2-Nal-OH and Fmoc-Oic-OH were purchased from AAPPTec (Luisville, KY, USA). Fmoc-Ser(*t*Bu)-OH, Fmoc-(Me)Phe-OH, Fmoc-Gly-OH, Fmoc-Pro-OHꞏH_2_O, Fmoc-Arg(Pbf)-OHꞏnIPE, Fmoc-Orn(Boc)-OH, Fmoc-Ahx-OH, and Fmoc-Ile-Alko resins were purchased from Watanabe Chemical Industries, Ltd. (Hiroshima, Japan). Tri-*tert*-butyl 1,4,7,10-tetraazacyclododecane-1,4,7,10-tetraacetate (DOTA(O*t*Bu)_3_) and 1-hydroxybenzotriazol (HOBt) were purchased from Nacalai Tesque, Inc. (Kyoto, Japan). 1,3-Diisopropylcarbodiimide (DIC) was purchased from FUJIFILM Wako Pure Chemical Co. (Tokyo, Japan). All reagents were used without further purification. Fmoc-Ahx-R954 was synthesised by solid-phase peptide synthesis using Fmoc-protected amino acids, as previously described [36]. After construction of the peptide chain on the resin, the Fmoc protecting group was removed by treatment with 20% piperidine in *N*,*N*-dimethylformamide (DMF), and a mixture of DOTA(O*t*Bu)_3_, DIC, and HOBt (2.5 equivalents each) in DMF was added and reacted for 3 h. Reaction completion was monitored using the Kaiser test. The protected peptide resin was treated with a mixture of 86.5% trifluoroacetic acid (TFA), 5% water, 5% phenol, 2.5% 1,2-ethanedithiole, and 1% triisopropylsilane at room temperature for 2 h, and then the resin was removed by filtration. After the filtrate was cooled to 0 °C, cold diethyl ether was added to precipitate the crude peptide, and the precipitate was washed twice with cold diethyl ether. The crude peptide was purified using preparative reverse-phase high-performance liquid chromatography (RP-HPLC) on a Cosmosil 5C_18_-AR-II column (10 mm inner diameter × 250 mm, Nacalai Tesque Inc.). The elution was carried out with a mixture of acetonitrile and water containing 0.1% TFA at a flow rate of 2.0 mL/min. The percentage of acetonitrile was maintained at 30% for the first 2 min and then increased in a linear gradient from 30 to 55% over 60 min. The eluate was monitored at 230 nm. Fractions containing the desired products were collected and lyophilised. The mass spectra of the purified peptides were acquired using a T100-LP (JEOL Ltd., Tokyo, Japan). HR-MS (ESI): *m/z* calcd. for C_81_H_123_N_18_O_19_ [M+H]^+^ 1651.9212, found 1651.9245; *m/z* calcd. for C_81_H_124_N_18_O_19_ [M+2H]^2+^ 826.4645, found 826.4635.

### 4.2. Radiolabelling

[^111^In]InCl_3_ (74 MBq/mL in saline) was purchased from Nihon Medi-Physics (Tokyo, Japan). A solution of [^111^In]InCl_3_ (14.1 MBq) in 0.02 N HCl (190 μL) was added to a solution of DOTA-Ahx-R954 (10 nmol) in a mixture of methanol (10 μL) and sodium acetate buffer (2.0 M, pH 4.5; 10 μL), and the mixture was heated on a heating block at 75 °C for 1 h. The reaction mixture was cooled to room temperature and purified using RP-HPLC, as described above (Section 4.1). The eluate was monitored using an in-line NaI(Tl) radiodetector (Gabi Star, Raytest, Straubenhardt, Germany). The desired radioactive peak was collected, neutralised with 1 M NaOH, concentrated to approximately 100 μL under vacuum, and diluted to 500 μL in saline. [^111^In]In-DOTA-Ahx-R954 was obtained with a radiochemical yield of 38.3% (5.39 MBq), a specific radioactivity of 5.43 GBq/μmol, and a radiochemical purity greater than 99%.

### 4.3. In Vitro Assay Using Glioblastoma Cells

Human glioblastoma cell lines U87MG and U251MG were obtained from the American Type Culture Collection (Manassas, VA, USA) and kept under standard cell culture conditions (5% CO_2_, 37 °C). Cells were cultured in Eagle’s minimum essential medium (E-MEM; FUJIFILM Wako Pure Chemical Co.) supplemented with 10% *v*/*v* foetal bovine serum (BioWest S.A.S, Nuaillé, France), 1% *v*/*v* nonessential amino acids (FUJIFILM Wako Pure Chemical Co.), 1 mM sodium pyruvate (FUJIFILM Wako Pure Chemical Co.), and 1% *v*/*v* penicillin–streptomycin (10,000 unit-10 mg/mL; FUJIFILM Wako Pure Chemical Co.). Cells were seeded at a density of 1 × 10^5^ cells/well in Poly-d-Lysine 24-well plates (Corning Inc., Corning, NY, USA). After 24 h, the medium was removed, and the cells were washed with 500 μL of 0.01 M PBS (+) (FUJIFILM Wako Pure Chemical Co.) and incubated at 37 °C in 500 μL of Hanks Balanced Salt Solution (+) (HBSS (+); FUJIFILM Wako Pure Chemical Co.) for 10 min. After preincubation, 250 μL of HBSS (+) containing [^111^In]In-DOTA-Ahx-R954 (3.5 nM) was added to each well, and incubation was continued. At 10, 30, 60, 120, and 180 min, the incubation solution was removed, and the cells were washed twice with 500 μL of ice-cold 0.01 M PBS (+) and solubilised in 500 μL of 0.3 M NaOH. The value at 0 min was determined by an assay performed using the same protocol as that used immediately after removing the incubation solution. Cell lysate radioactivity was measured using an auto-well gamma counter (2470 WIZARD^2^; PerkinElmer, Inc., Shelton, CT, USA), and the protein content of the lysates was measured using a Pierce™ BCA protein assay kit (Thermo Fisher Scientific, Inc., Waltham, MA, USA). The results are presented as the percentage dose per milligram of protein (% dose/mg protein).

To examine the specific uptake of [^111^In]In-DOTA-Ahx-R954, cells were incubated with [^111^In]In-DOTA-Ahx-R954 in the presence of 100 μM B1R antagonist R954 (pIC_50_ = 10.0 ± 3.1 nM) or B1R agonist [des-Arg^10^]-kallidin (pEC_50_ = 9.7 nM) [37,38] for 120 min. The affinity of [^111^In]In-DOTA-Ahx-R954 for B1R in the cells was measured using an R954 saturation assay with increasing concentrations of [^111^In]In-DOTA-Ahx-R954 (range 0.7–358 nM). The Scatchard plot was used to estimate the K_d_ and B_max_ values. The parameters were presented as means of three independent experiments (one assay performed on type I collagen-coated plates and two assays performed on Poly-d-Lysine plates).

### 4.4. Western Blotting

Cell lysates were prepared from cultured U87MG and U251MG cells by homogenisation in ice-cold RIPA buffer (Cell Signaling Technology, Inc., Danvers, MA, USA) containing a Protease Inhibitor Cocktail (Merck KGaA, Darmstadt, Germany). The protein content of the lysates was determined using the BCA method, and 20 μg of protein per lane was subjected to electrophoresis on 5–20% sodium dodecyl sulphate-polyacrylamide gels and then transferred onto a nitrocellulose membrane. After blocking with TBST buffer (20 mmol/L Tris–HCl, 150 mmol/L NaCl, 0.5 mL/L Tween 20) containing 5% skim milk, the membranes were incubated with a rabbit polyclonal anti-human BDKRB1 primary antibody (Proteintech Group, Inc., Rosemont, IL, USA). After repeated washes, the membranes were incubated with an Amersham™ horseradish peroxidase-conjugated anti-rabbit secondary antibody (Cytiva, Marlborough, MA, USA). Immunoreactive bands were detected using Chemi-Lumi One Ultra (Nacalai Tesque Inc.) and an Omega Lum G luminescent image analyser (Aplegen Inc., Pleasanton, CA, USA). The protein-loading control for immunoblotting was β-actin, which was detected by a mouse monoclonal anti-β-actin-peroxidase antibody (Merck KGaA).

### 4.5. Animal Model

All experimental protocols were approved by the Animal Care and Use Committee of Showa Pharmaceutical University and performed in accordance with the Principles of Laboratory Animal Care. Five-week-old BALBc nu/nu male mice (Japan SLC, Inc., Hamamatsu, Japan) were xenografted by subcutaneous injection of U87MG cells (5 × 10^6^ cells per 50 μL of culture medium) into their right hind legs. The mice were subjected to biodistribution studies when the tumour weight reached 0.1−0.5 g.

### 4.6. Biodistribution in Glioblastoma-Bearing Mice

The experimental conditions, including the dosage of [^111^In]In-DOTA-Ahx-R954, were in line with the literature on ^111^In-labelled peptide [39] and modified based on pre-experiments. Mice bearing U87MG tumour xenografts were injected via the tail vein with 100 μL of [^111^In]In-DOTA-Ahx-R954 (30–74 kBq; 0.01–0.02 nmol) and were sacrificed and dissected 1, 4, and 24 h after administration. For the blocking experiments, 100 μg of R954 was used, which was the same amount used for ^68^Ga-HTK01083 by Kuo et al. [17]. R954 was co-injected with [^111^In]In-DOTA-Ahx-R954, and mice were sacrificed and dissected 1 and 4 h after administration. The tissues of interest were removed and weighed, and radioactivity was determined using an auto-well gamma counter. The results are presented as a percentage of the injected dose per gram (% dose/g).

### 4.7. Ex Vivo Autoradiography

Double tracer autoradiography using [^111^In]In-DOTA-Ahx-R954 and [^14^C]iodoantipyrine was performed as previously described [40]. Mice were intravenously injected with [^111^In]In-DOTA-Ahx-R954 (74 kBq), whereas [^14^C]iodoantipyrine (37 kBq, PerkinElmer, Inc.) was injected 59 min after the [^111^In]In-DOTA-Ahx-R954 injection. Mice were decapitated 1 min after the injection of [^14^C]iodoantipyrine, and the tumour and muscle tissues were quickly removed and frozen on dry ice. Sections (50 μm) were prepared using a cryostat (Leica CM1520, Leica Biosystems, Deer Park, IL, USA) and exposed to an imaging plate (Cytiva) for 20 h to obtain [^111^In]In-DOTA-Ahx-R954 images. After the decay of ^111^In, the same sections were re-exposed to an imaging plate for three days to obtain [^14^C]iodoantipyrine images. Autoradiograms were obtained using the Phosphor Imaging Digitize system of an Amersham™ Typhoon™ scanner (Cytiva). Regions of interest (ROIs) were created on the images, and the radioactivity concentration in each ROI was expressed as photostimulated luminescence ([PSL minus background]/area [mm^2^]).

### 4.8. Immunohistochemistry

Consecutive tumour and muscle slices from the same object used in the ex vivo autoradiographic study were used for immunohistochemical analysis. The primary antibodies used in this study were rabbit polyclonal anti-human BDKRB1 (LifeSpan BioSciences, Inc., Seattle, WA, USA) and mouse monoclonal anti-GFAP (Merck KGaA). The sections were fixed with acetone:methanol (1:1) for 20 min at −20 °C. After antigen retrieval using HistoVT One (Nacalai Tesque Inc.), sections were blocked with Blocking One Histo (Nacalai Tesque Inc.) for 10 min at 25 °C. After overnight incubation of the sections with the mixed antibodies at 25 °C, the primary antibodies were visualised using either an Alexa Fluor 488-labelled anti-rabbit IgG (H+L) secondary antibody (Thermo Fisher Scientific Inc.) or a Cy3-labelled anti-mouse IgG (H+L) secondary antibody (Jackson ImmunoResearch Labs Inc., West Grove, PA, USA). Fluorescence images were captured using a fluorescent microscope (BX50, Olympus, Tokyo, Japan). To evaluate the distribution of GFAP immunoreactive cells in whole tissue sections, we obtained fluorescence images detected by a 532-nm excitation laser and a 580-nm long-pass detection filter with an Amersham™ Typhoon™ scanner. The fluorescence intensity in the slices was determined as linear arbitrary units (LAUs) corrected for background ([LAU-background]/area [mm^2^]) using Multi Gauge Analysis Software version 3.11 (Fuji Film Co., Tokyo, Japan).

### 4.9. Statistical Analysis

All values were expressed as the mean ± SD (for each group). According to the Student paired or unpaired *t*-tests, statistical significance was set at *p* < 0.05.

## 5. Conclusions

In this study, we demonstrated that [^111^In]In-DOTA-Ahx-R954, a radiolabelled derivative of the B1R antagonist, can be used to detect the endogenous expression of B1R in glioblastoma cells. Our results suggest that radiolabelled probes targeting B1R are potentially valuable tools for the detection of differentially expressed B1R. This versatility is essential for expanding its applicability across various cancer types and allowing for the clinical translation of B1R imaging in the future.

## Figures and Tables

**Table 1:** Tissue distribution of [^111^In]In-DOTA-Ahx-R954 in U87MG-bearing mice.

	1 h	4 h	24 h
Radioactivity (% Dose/g)			
Plasma	2.75 ± 0.26	0.06 ± 0.03	0.04 ± 0.01
Heart	0.83 ± 0.16	0.29 ± 0.05	0.21 ± 0.03
Lung	2.37 ± 0. 97	0.42 ± 0.07	0.26 ± 0.08
Liver	1.57 ± 1.36	2.36 ± 0.31	1.18 ± 0.27
Kidney	120.1± 19.9	159.3 ± 8.76	109.2 ± 22.9
Stomach	0.79 ± 0.09	0.31 ± 0.09	0.18 ± 0.04
Small Intestine	0.68 ± 0.02	0.36 ± 0.04	0.22 ± 0.03
Large Intestine	0.34 ± 0.04	0.69 ± 0.29	0.46 ± 0.13
Pancreas	0.55 ± 0.06	0.28 ± 0.07	0.22 ± 0.06
Spleen	0.55 ± 0.10	0.35 ± 0.07	0.36 ± 0.08
Bone	0.60 ± 0.32	0.50 ± 0.27	0.42 ± 0.20
Brain	0.04 ± 0.003	0.02 ± 0.013	0.01 ± 0.004
Muscle	0.61 ± 0.10	0.41 ± 0.04	0.32 ± 0.07
Tumour	1.37 ± 0.06 *	0.71 ± 0.09 *	0.68 ± 0.12 *
Tumour/tissue ratios of radioactivity			
Tumour/Plasma	0.50 ± 0.03	13.12 ± 4.98	19.00 ± 3.53
Tumour/Muscle	2.31 ± 0.42	1.72 ± 0.16	2.15 ± 0.35

Data are expressed as mean ± SD (in 1 h, *n* = 3, and in others, *n* = 4). * *p* < 0.01, Muscle vs. Tumour.

**Table 2:** Effects of simultaneous administration of R954 on the tissue uptake of [^111^In]In-DOTA-Ahx-R954 in U87MG-bearing mice.

	1 h		4 h	
	Vehicle	R954	Vehicle	R954
Radioactivity (% Dose/g)				
Plasma	2.00 ± 0.58	7.49 ± 1.68 ***	0.15 ± 0.09	0.50 ± 0.13 **
Heart	0.55 ± 0.20	1.45 ± 0.36 **	0.32 ± 0.06	0.36 ± 0.07
Lung	1.16 ± 0.30	3.49 ± 1.12 **	0.46 ± 0.16	0.53 ± 0.17
Liver	1.65 ± 0.33	2.82 ± 0.55 **	2.25 ± 0.68	2.99 ± 0.77
Kidney	67.2 ± 14.1	26.8 ± 13.7 **	111.0 ± 13.45	92.2± 9.86 *
Stomach	0.54 ± 0.09	1.70 ± 0.54 **	0.55 ± 0.12	0.52 ± 0.26
Small Intestine	0.58 ± 0.18	1.22 ± 0.54 *	0.54 ± 0.18	0.72 ± 0.32
Large Intestine	0.27 ± 0.08	0.65 ± 0.21 **	1.06 ± 0.60	1.50 ± 0.65
Pancreas	0.37 ± 0.09	0.85 ± 0.19 ***	0.29 ± 0.05	0.28 ± 0.10
Spleen	0.36 ± 0.08	0.96 ± 0.24 ***	0.42 ± 0.12	0.51 ± 0.21
Bone	0.44 ± 0.16	1.30 ± 0.26 ***	0.49 ± 0.28	0.58 ± 0.19
Brain	0.03 ± 0.01	0.09 ± 0.03 **	0.02 ± 0.01	0.03 ± 0.01
Muscle	0.37 ± 0.08	1.21 ± 0.17 ***	0.42 ± 0.06	0.31 ± 0.10
Tumour	0.82 ± 0.25	2.13 ± 0.48 ***	0.66 ± 0.11	0.72 ± 0.10
Tumour/tissue ratios of radioactivity				
Tumour/Plasma	0.41 ± 0.03	0.29 ± 0.03 ***	4.89 ± 1.27	1.51 ± 0.33 ***
Tumour/Muscle	2.18 ± 0.24	1.76 ± 0.30 *	1.57 ± 0.24	2.56 ± 1.05

Data are expressed as mean ± SD (*n* = 5). * *p* < 0.05, ** *p* < 0.01, *** *p* < 0.001 vs. Vehicle.

## Data Availability

Data are contained within the article or Supplementary Materials.

## Supplementary Materials

The following supporting information can be downloaded at: https://www.mdpi.com/article/10.3390/ph17070902/s1, Figure S1: High-resolution electrospray ionization mass spectra of DOTA-Ahx-R954; Figure S2: RP-HPLC chromatogram of [^111^In]In-DOTA-Ahx-R954; Figure S3: Extended data from the western blot analysis of B1R shown in Figure 2A; Figure S4: Representative TLC chromatograms of mouse plasma samples 60 min after the injection of [^111^In]In-DOTA-Ahx-R954.

## Abbreviations

bradykinin receptor B1 (B1R); bradykinin receptor B2 (B2R); 1,3-diisopropylcarbodiimide (DIC); *N*,*N*-dimethylformamide (DMF); Eagle’s minimum essential medium (E-MEM); glial fibrillary acidic protein (GFAP); 1-hydroxybenzotriazol (HOBt); linear arbitrary units (LAUs); photostimulated luminescence (PSL); positron emission tomography (PET); region of interest (ROI); reverse-phase high-performance liquid chromatography (RP-HPLC); single-photon emission computed tomography (SPECT); tri-*tert*-butyl 1,4,7,10-tetraazacyclododecane-1,4,7,10-tetraacetate (DOTA(OtBu)_3_); trifluoroacetic acid (TFA).
