# Supplementary material for: Assessment of Radiolabelled Derivatives of R954 for Detection of Bradykinin B1 Receptor in Cancer Cells: Studies on Glioblastoma Xenografts in Mice
Source: Pharmaceuticals (Basel). 2024 Jul 7;17(7):902. doi: 10.3390/ph17070902 (PMC11279923; doi:10.3390/ph17070902)
Supplement: Supplementary file 1 [file pharmaceuticals-17-00902-s001.zip › pharmaceuticals-3090945-supplementary.pdf]

# Supplemental Information

## Supplemental Figures

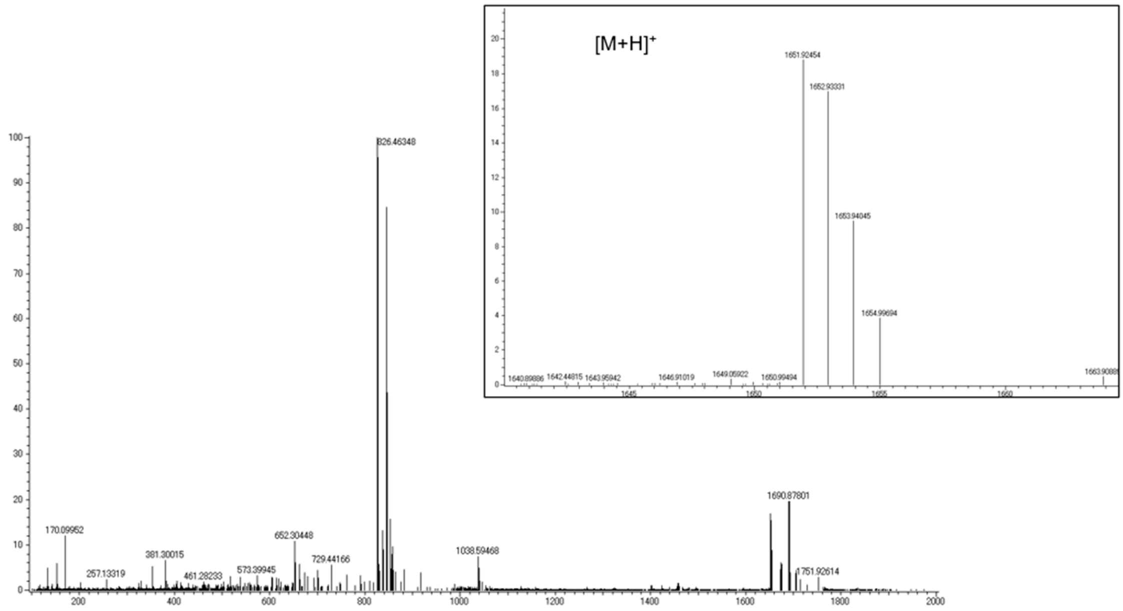


**Figure S1.** High-resolution electrospray ionisation mass spectra of DOTA-Ahx-R954. Inset: expansion to show isotopic pattern.

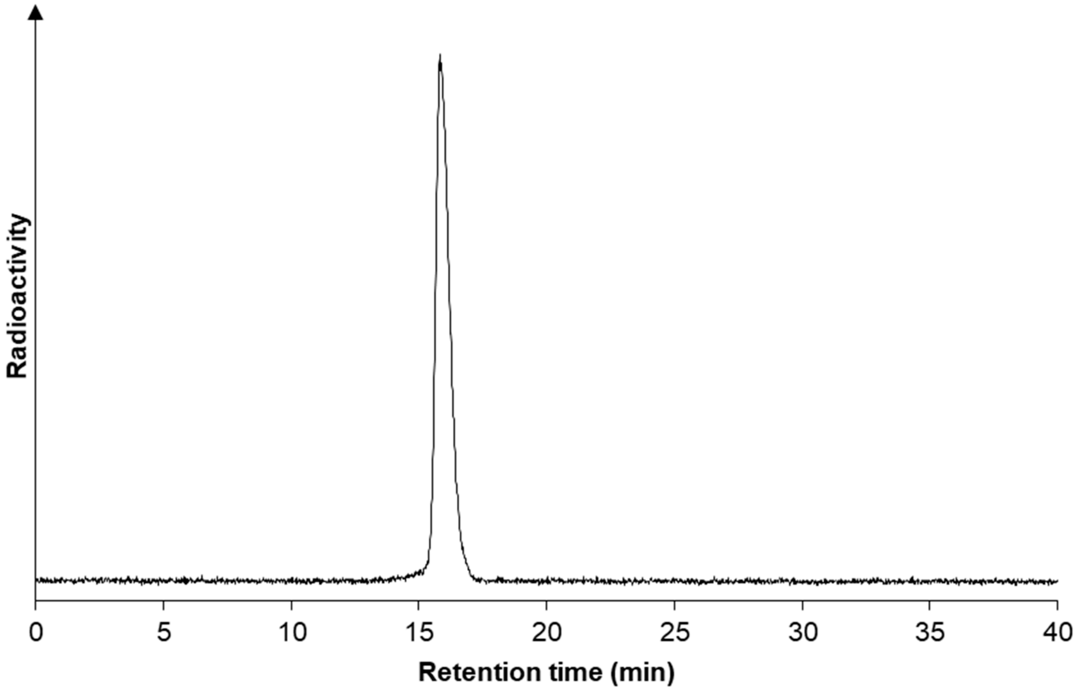


**Figure S2.** RP-HPLC chromatogram of [$^{111}$In]In-DOTA-Ahx-R954.

(A)

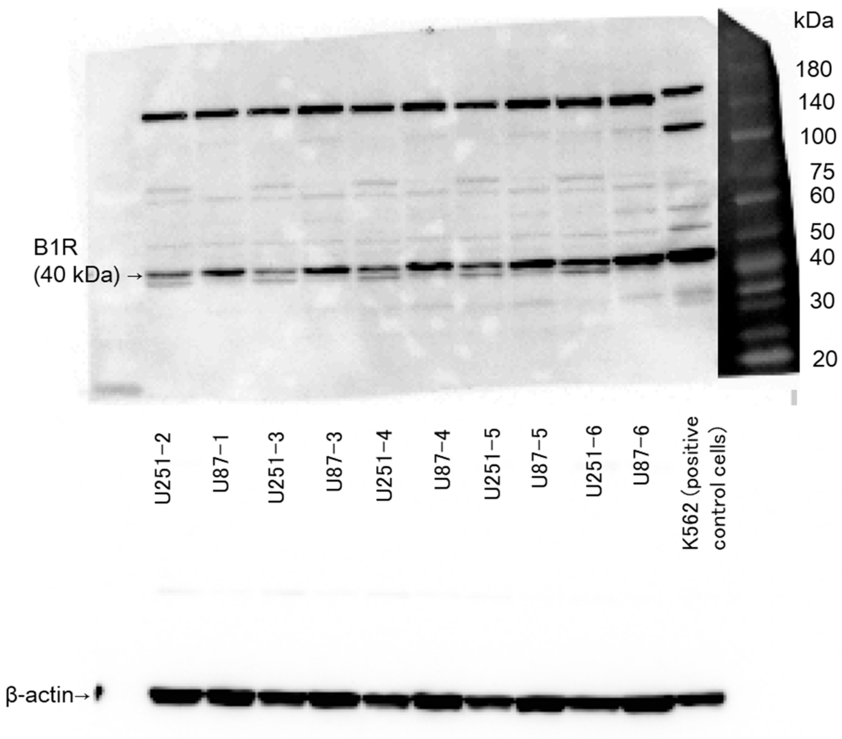


(B)

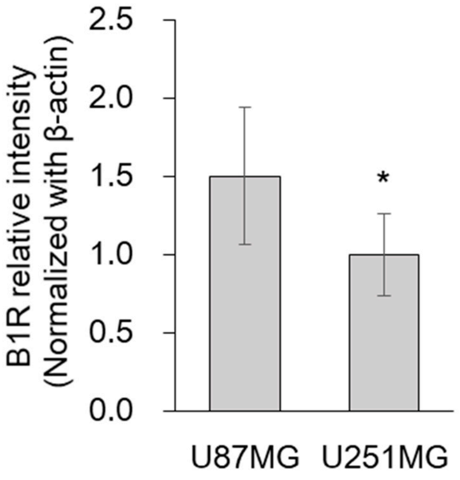


**Figure S3.** Extended data from the western blot analysis of B1R shown in Figure 2-A. (**A**) Uncropped western blot images; (**B**) Quantitative analysis of relative intensity of B1R expression. The values represent the signal intensity of protein bands in arbitrary units after normalisation with the signal intensity of β-actin internal control for each sample. Data were collected from three independent experiments and presented as means ± SD. * $p < 0.001$, U87MG vs. U251MG.

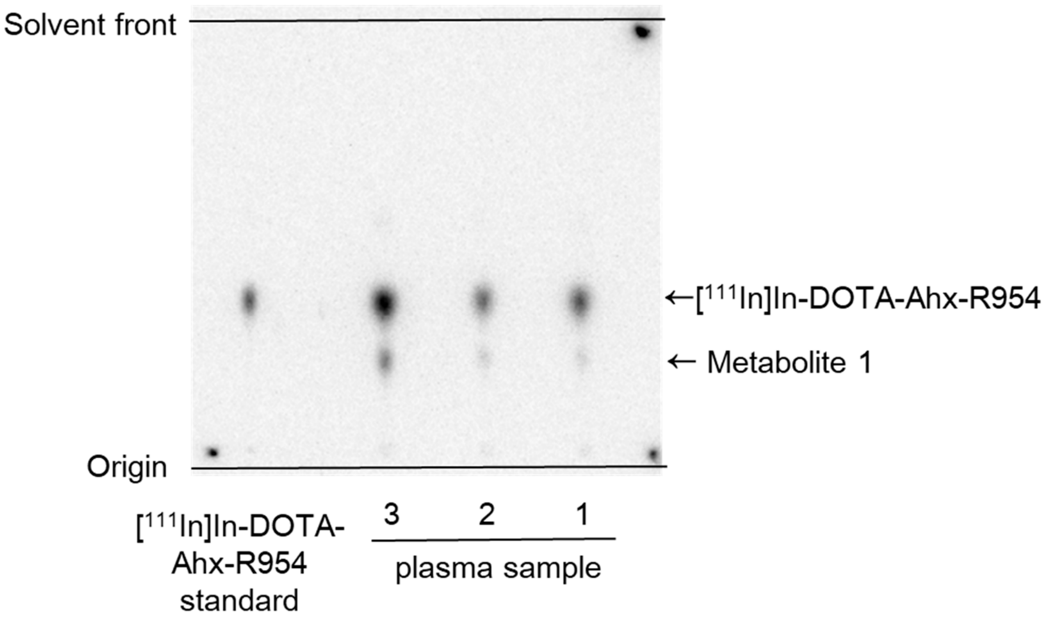


**Figure S4.** Representative TLC chromatograms of mouse plasma samples 60 min after the injection of [$^{111}$In]In-DOTA-Ahx-R954. One radiometabolite (Metabolite 1) was observed, and 79.6 ± 3.1% of radioactivity remained intact ([$^{111}$In]In-DOTA-Ahx-R954) in plasma.

## Supplemental Methods

*Metabolite analysis of mouse plasma samples*

Blood samples were obtained from C57BL/6JSlc male mice (6 weeks old, Japan SLC, Inc., Hamamatsu, Japan) at 60 min following the injection of 74 kBq of [$^{111}$In]In-DOTA-Ahx-R954. The blood was spun at 12,000 rpm for 5 min, and two-fold volumes of acetonitrile were added to the aliquot of plasma. The resulting mixture was centrifuged at 12,000 rpm for 2 min at 4 °C. The aliquot (20 μL) of the supernatant was analysed using TLC Silicagel 60 F254 plates (Merck KGaA). Plates were developed with methanol/10% ammonium chloride (50/50, v/v) as the mobile phase. Dried plates were exposed using an imaging plate overnight and were imaged using an Amersham™ Typhoon™ scanner.
